# Book Notices

**Published:** 1880-09-20

**Authors:** 


### *BOOK NOTICES.*

TRANSACTIONS OF THE MISSISSIPPI STATE MEDICAL ASSOCIATION, at the 13th Annual Session held at Vicksburg, April 7, 8 and 9, 1880, with the roll of Members and reports on medical topics —published by the Association.

The present number of the Mississippi Transactions is one of unusual interest. It is neatly gotten up, and contains 177 octavo pages.

The Address of President E. P. Sale, M.D., on the duties we owe our women, is one of marked ability and interest; so is the Annual Oration, by B. F. Ward, M.D.

We note also as worthy of commendation the following interesting papers:

Recent Advances in Surgery, By S. V. D. Hill, M.D.

Recent Advances in Obstetrics, by N. L. Guice, M.D.
Ascyrum Crux-Andreæ, for Pertussis, by D. L. Phares, M.D.
Malarial Hematuria, by J. M. Greene, M.D.
Hemorrhagic Malarial Fever, by B. F. Kittrel, M.D.
Treatment of Wounds, by C. A. Rice, M.D.
Strangulated Inguinal Hernia, by John Brownrigg, M.D.
Amputation of Both Legs, by Wirt Johnson, M.D.
A Case of Traumatic Tetanus, by Wirt Johnson, M.D.
Notes on some Cases of interest, by W. A. Taylor, M.D.
Removal of Urethral Calculi by Perineal Section, by J. E. Halbert, M.D.
A Case in Practice, by John Featherston, M.D.
A Case of Lithotomy, by J. W. Bennett, M.D.
Congenital Malformation of the Anus, by J. C. Hall, M.D.
Simple Uterine and Vaginal Irrigator, by John S. Featherston, M.D.
Case of Atresia Vaginæ, complicated with Pregnancy, by E. P. Sale, M.D.
Yellow Fever at Concordia, by John B. Pease. M.D.

The following are the Officers elect for the ensuing year:

*President.*—W. F. HYER, M.D., Hudsonville.

*Vice-Presidents.*—1st. D. L. Phares, M.D., Woodville. 2d. H. Shannon, M.D., Vicksburg. 3d. R S. Toombs, M.D., Greenville. 4th. W. D. Carter, M.D, Ripley.

*Recording Secretary.*—Wirt Johnson, M.D., Jackson.

*Corresponding Secretary.*—M. S. Craft, M.D., Jackson.

*Treasurer.*—Geo. K. Harrington, M.D., Jackson.

*Orator.*—S. D. Robbins, M.D., Vicksburg.

*Alternate Orator.*—S. R. Dunn, M.D., Greenville.

A PRACTICAL TREATISE on Tumor of the Mammary Gland, embracing their Histology, Pathology, Diagnosis and Treatment, by Samuel W. Gross, A.M., M.D., Surgeon to and Lecturer on Clinical Surgery in the Jefferson Medical College Hospital and the Philadelphia Hospital, President of the Pathological Society of Philadelphia, Fellow of and former Master Lecturer on Surgical Pathology in the College of Physicians of Philadelphia, Fellow of the Academy of Surgery of Philadelphia, etc. Illustrated by twenty-nine engravings. 246 pages octavo.—New York, D. Appleton & Co., 1, 3 and 5, Bond Street, 1880.

This contribution from Dr. Gross fills a void in our medical literature. The author has brought his extensive knowledge and powers of research into requisition in the investigation of the pathology, histological character and differential diagnosis of mammary diseases, including carcinoma, etc. He holds that cancer may be permanently relieved by thorough operations practiced in the early stage of its evolution. He brings many facts to sustain this position.

The work contains much that is new and instructive, and will be gladly welcomed by the profession.

SUPPLEMENT TO THE AMERICAN DISPENSATORY, by John King, M.D., Professor of Obstetrics and Diseases of Women and Children in the Eclectic College of Medicine, Cincinnati, of Materia Medica, Therapeutics and Medical Jurisprudence, etc., etc.; and by John U. Lloyd, Professor of Chemistry and Pharmacy in the Eclectic Medical Institute of Cincinnati, etc.—Witstach, Baldwin & Co., 143 Race Street, Cincinnati.

This is an eclectic work, and so the American Dispensatory to which it is a Supplement. We would not be understood in this review as endorsing that system, but as a journalist seek to give our readers information upon all subjects which the facilities of our position throw into our hands.

The interest now taken in the department of Therapeutics, and the advances which are being made have developed an eager desire to examine and test the new remedies from whatever source they come.

The work or Supplement now under review is devoted in the main to a history, description, and therapeutical properties of new remedies. It contains much that is new and interesting; the illustrations are original and are life-like and true to nature.

Prof. J. U. Lloyd was intrusted with this department of the work and states that "The chemical and pharmaceutical processes given are derived in a large degree from personal experience in laboratory work." As a chemist and practical pharmaceutist, Prof. Lloyd stands deservedly high.

THE SKIN IN HEALTH AND DISEASE, by L. Duncan Bulkley, M.D., Attending Physician for Skin and Venereal Diseases at the New York Hospital, Out Patient Department; Late Physician to the Skin Department, Dermatological Dispensary, New York, etc.—Philadelphia, Presley Blakiston, 1012 Walnut Street, 1880.

This is a neat little work of 142 pages, embracing chapters on the following general heads: Anatomy and Physiology of the Skin; the Care of the Skin in Health; Diseases of the Skin; Diet and Hygiene in Diseases of the Skin.

THF BRAIN AS AN ORGAN OF THE MIND, by H. Charlton Bastian, M.A., M.D., F. R. S., Professor of Pathological Anatomy and of Clinical Medicine in University College, London, Physician to University College Hospital and to the National Hospital for the Paralyzed and Epileptic; with one hundred and eighty-four illustrations.—New York, D. Appleton & Co., 5 Bond Street, 1880.

This is a book of 700 pages, embracing recent views and discoveries in regard to the origin and functions of the nervous system, and containing many interesting and instructive facts and principles relative to the brain and mental operations. The chapters upon speech and thought, and the localization of the higher cerebral functions, we perused with special interest, also that upon the nature and origin of instinct. It is a valuable and instructive work, and will repay perusal.

